# Exploring the impact of mental and work-related stress on sick leave among middle-aged women: observations from the population study of women in Gothenburg, Sweden

**DOI:** 10.1080/02813432.2024.2380925

**Published:** 2024-07-29

**Authors:** Kirsten Mehlig, Amanda von Below, Kristina Holmgren, Cecilia Björkelund, Lauren Lissner, Ingmarie Skoglund, Magnus Hakeberg, Dominique Hange

**Affiliations:** a School of Public Health and Community Medicine, Institute of Medicine, Sahlgrenska Academy, University of Gothenburg, Sweden; b General Practice/Family Medicine, School of Public Health and Community Medicine, Institute of Medicine, Sahlgrenska Academy, University of Gothenburg, Gothenburg, Sweden; c Unit of Occupational Therapy, Department of Health and Rehabilitation, Institute of Neuroscience and Physiology, Sahlgrenska Academy, University of Gothenburg, Gothenburg, Sweden; d Research, Education, Development & Innovation, Primary Health Care, Region Västra Götaland, Sweden; e Institute of Odontology, Sahlgrenska Academy, University of Gothenburg

**Keywords:** Mental stress, population study, sick leave, women, work-related stress

## Abstract

**Objective:**

To investigate whether mental and work-related stress predicts a one-year incidence of sick leave in a cohort of middle-aged working women.

**Design:**

The 2016/17 survey was part of the Population Study of Women in Gothenburg, Sweden, with registry data information on sick leave during one year pre- and post-baseline.

**Subjects:**

A cohort of women aged 38 and 50 in 2016/17 (*n* = 573; 68% participation), of which 504 women were gainfully employed and not on sick leave ± 2 weeks around baseline examination; 493 women had complete data on stress exposure.

**Methods:**

We studied associations between self-assessed mental and work-related stress and incident sick leave of >14 days during the year following the baseline examination. We used multiple logistic regression, adjusting for age and previous sick leave, and additionally for sleep quality, well-being, and physical activity.

**Results:**

Overall, 75 women (16%) experienced at least one period of sick leave after baseline. Permanent stress during the last five years almost tripled the risk for incident sick leave, OR = 2.8 (95% CI 1.2–6.3), independent of previous sick leave, OR = 2.3 (95% CI 1.3–4.2). Among 21 specific work-related problems, conflicts at work, OR = 2.2 (95% CI 1.3–3.6), and low decision latitude, OR = 1.7 (95% CI 1.0–2.9), were associated with incident sick leave. The association with conflicts at work remained upon further covariate adjustment.

**Conclusion:**

Low decision latitude and conflicts at work are risk factors for incident sick leave among working women. The impact of conflicts at work, irrespective of own involvement, may indicate a specific vulnerability among women of interest for future interventions.

## KEY POINTS

About 75% of around 500 middle-aged women reported mental stress in 2016/17. Low decision latitude and conflicts at work predicted incident sick leave independent of general mental stress and previous periods of sick leave. Efforts to improve the work environment may be essential for interventions aiming to reduce absenteeism among working women.

## Introduction

Mental stress is a subjective symptom with almost as many meanings as the number of people affected by it. As a biological concept, it was first defined by Hans Selye over 80 years ago as a ‘non-specific response of the body to any demand for change’ [1]. According to two more recent definitions by the American Institute of Stress, stress is the ‘physical, mental, or emotional strain or tension’ or ‘a condition or feeling experienced when a person perceives that demands exceed the personal and social resources the individual can mobilise’ [2].

Mental stress is a widespread issue internationally, not least since the COVID-19 pandemic [3–5]. It has previously been shown in the Population Study of Women in Gothenburg that mental stress doubled in frequency between 1968 and 2004 [6], and the trend continues as National surveys show that impaired psychological well-being and anxiety increased in the last 15 years, especially among younger women [7].

Mental stress can significantly impact a person’s well-being and ability to work. A recent systematic review highlighted that poor working conditions increased the risk of stress-related mental disorders [8] and subsequent sick leave [9,10]. Experiences of work-related stress have been related to sick leave in a working population seeking primary care [11,12].

In Western countries, women are more likely to seek help for common mental disorders than men [7,13,14], which translates into a notable disparity in sickness absence [15]. The gender difference might be related to factors such as caregiving responsibilities, discrimination and other factors at the workplace. The labour market is strongly segregated by gender, which means that women and men are working in different occupations and sectors. Larger proportion of women work in the welfare sector, resulting in a work environment that differ from the typically male work sector. This gender segregation is found to be associated with a higher risk for sick leave for both women and men especially in female-dominated occupations [16], but studies on specifically female work conditions are rare [17].

Sick leave, especially long-term sick leave exceeding 14 days, is an important issue in Sweden. There was an overall increase in sick leave in the first half of the 2010s, primarily attributable to increased anxiety- and stress-related diseases, e.g. burnout [18]. Risk factors associated with long-term sick leave in women are age, obesity, taking primary responsibility for own children/family, having more than two children, the experience of bullying at work, high mental strain and low influence at work [11,19–21].

This study is designed to address two key objectives. First, we aimed to investigate whether self-reported general mental stress and specific aspects of work-related stress predict incident episodes of sick leave in a contemporary Swedish cohort of middle-aged working women, and which of these two risk factors was more important with respect to sick leave.

The second objective was to identify potential confounders that may influence the associations with general and work-related stress. To achieve this, a set of covariates has been included: demographic variables such as education and socioeconomic status, health-related variables such as sleep quality and well-being, lifestyle variables comprising leisure-time physical activity, smoking habits, and alcohol consumption. We expect that our results will guide the development of effective interventions to reduce the incidence of sick leave in the Swedish female population and its impact on women’s mental health.

## Material and methods

### The Population Study of Women in Gothenburg (PSWG)

In 2016–17, a random sample of 1038 women aged 38 and 50 years and living in Gothenburg received a letter of invitation to the PSWG. Of these, 843 responded, 573 participated in the health examination.

Participation rates were 63% among the 38-year-old women and 73% among the 50-year-old women [22]. This survey is part of a long-term prospective study of health trends in Swedish women, with earlier-born cohorts of 38- and 50-year-olds recruited in similar surveys between 1968 and 2004 [23].

### Periods of sick leave

Data regarding sick leave (>14 days) were obtained from the Micro Database for Analysing Social Insurance (MiDAS) [24]. The cohort examination took place between 17 October 2016 and 31 May 2017, and all periods of sick leave starting, ending or ongoing between 17 October 2015 – 31 May 2018 were included in this analysis. Depending on the participants’ examination date, follow-ups varied between one year and 591 days, with an average of 470 days. For this study, we defined the following binary variables: having had any periods of sick leave that ended ≥ two weeks before the baseline examination (previous sick leave), being on sick leave within ± two weeks of the baseline examination (current sick leave), and being on sick leave ≥ two weeks after the baseline examination (incident sick leave). The gap period before the examination ensures that the women have worked a certain time after sick leave to be able to give a proper account of work-related stress at the examination. The gap period after baseline may reduce the risk that the self-reported stress at baseline is a consequence of imminent sick leave, rather than a risk factor for it.

### Mental stress

General mental stress was defined as one or several periods of irritability, tension, nervousness, anxiety, anxiousness or insomnia due to work, health, family or conflicts at work or home. Response alternatives were the following: 1. Never experienced mental stress, 2. Experienced mental stress, but not during the last five years, 3. Occasionally experienced mental stress during the last five years, 4. Experienced mental stress several times during the last five years, 5. Experienced mental stress constantly during the last year, 6. Experienced mental stress constantly during the last five years. These items were combined into a three-level categorical variable distinguishing no mental stress during the last five years (level 1 + 2), some periods of stress during the last five years (level 3 + 4) and permanent stress during the last five years (level 5 + 6). The mental stress question is comprehensive and covers various aspects of stress and has been shown to be e.g. associated to cancer [25] and dementia [26] and has been consistently used in an identical form across all examinations between 1968 and 2016–17.

### Work-related stress

Work-related stress was assessed using the Work Stress Questionnaire (WSQ) [27,28], a self-assessed instrument consisting of 21 main questions grouped into four domains; i.e. *influence at work*; *indistinct organisation and conflicts*; *individual demands and commitment;* and *interference with leisure time. Influence at work* and *interference with leisure time* include four and three questions, respectively, and answers are given on a four-point ordinal scale; Yes, always; Yes, rather often; No, seldom and No, never. Answers to these questions are scored as 1-4, with higher values indicating more problems. The other two domains contain seven questions each with three possible answers: Yes; Partly and No. If Yes or Partly is chosen, participants are asked to answer the follow-up question ‘Do you perceive the problem as stressful?’, with possible response categories given by Not at all stressful; Less stressful; Stressful and Very stressful. As previously, the answers are scored as 1-4 with a score equal to one given if no problem is identified, or if the problem in question does not cause any stress.

Domain scores were calculated by computing the median values of the appropriate items. Both domain as well as individual question scores were then dichotomised at the median value, and the percentage of women experiencing the stressor (i.e. with scores > median) was calculated. The reliability and face validity of the WSQ have been rigorously assessed to ensure the robustness of the instrument [25,26]. In the present study, internal consistency was evaluated using Cronbach’s alpha, yielding satisfactory results across different domains. Specifically, Cronbach’s alpha ranged from 0.64 for the domain of *influence at work,* 0.80 for *indistinct organisation and conflicts*, 0.88 for *individual demands and commitment* to 0.90 for *interference with leisure time*.

### Potential confounders

Anthropometric measures were taken following standard protocols [23]. Body mass index (BMI) was calculated as weight/length^2^ (kg/m^2^). The waist-hip ratio (WHR) was calculated as waist circumference divided by hip circumference. The participant’s highest educational level was categorised as university education versus less. A socioeconomic index (SEI) was estimated based on the maximum of the woman’s and her partner’s employment and categorised as high, medium and low [29]. The women reported whether they were living with a partner or not, and whether they had children living at home or outside home. The women reported their previous and current smoking habits, which were dichotomised as current vs. former or never smoking. The participants were asked about the frequency of habitual consumption of beer, wine and liquor, and the frequency of any alcoholic beverage was calculated (times/week). Information about the participants’ leisure time physical activity (LTPA) during the last year was obtained distinguishing sedentary behaviour, moderate activity, regular activity and competitive sports. Moderate activity was defined as walking or cycling for at least four hours per week. Regular activity included running, dancing, swimming, playing tennis or heavy gardening on a weekly basis. Competitive sports included hard physical training several times per week. Participants rated their sleep quality and well-being using the Gothenburg Quality of Life Instrument [30]. The instrument utilises a seven-point Likert-type scale with response categories ranging from 1 (Excellent, couldn’t be better) to 7 (Very poor).

### Definition of the study sample

Among the 573 women who participated in the examination in 2016–17, 33 women were excluded because they were not currently working due to e.g. disability pension, unemployment or parental leave, and 36 women were excluded because they were on sick leave during the two weeks before or after the baseline examination. The exclusion of women on sick leave ± two weeks around baseline ensures that women were actively working when reporting on work-related stress and reduces the risk that self-reported stress exposure is a consequence of imminent sick leave rather than a risk factor for it. Two women were excluded because they did not complete the question about mental stress, leaving 502 women for the analysis of mental stress and incident sick leave. Nine other women had incomplete information on work-related stress, leaving 493 women for association analyses involving work-related stress (See flowchart in Table S1).

### Statistical method

The Kruskal-Wallis test was used to compare continuous variables by self-reported stress level, and the chi-square test was used for categorical variables. The Cochran-Armitage test was used for trends in binary variables across levels of increasing stress. Ordinal logistic regression with stepwise variable selection was used to determine correlates of self-reported stress that has three ordered categories. This model assumes that covariate effects on adjacent outcome levels are the same regardless of reference level, i.e. permanent vs. some stress or some vs. no stress. A non-significant result for the score test of the proportional odds assumption indicated the validity of the model [31]. As a result, odds ratios (OR) with a 95% confidence interval (CI) were given for the selected covariates. Associations between categories of mental stress and incident sick leave were tested in a multivariable logistic model adjusting for age and previous sick leave (model 2) as well as covariates of mental stress, i.e. well-being, sleep quality, and LTPA (model 3). Depending on the date of their baseline examination women had different follow-up times until May 31, 2018. For this reason, all regression models were also adjusted for the date of examination. Sensitivity analyses were performed with identical follow-up for all women, i.e. one year after their baseline examination. Regarding work-related stress, we dichotomised each WSQ item and the four domain scales at their respective median. The proportion of participants with values above the median was compared across categories of mental stress using the Cochran-Armitage test of trend. We defined an age-adjusted logistic regression with stepwise variable selection applied to the set of single WSQ items and the four domain scales to obtain the most important predictors of incident sick leave (model 1). The model was further adjusted for previous sick leave and mental stress (model 2), and covariates of mental stress (model 3). The prediction accuracy of logistic regression models was assessed using the area under the ROC curve (AUROC). All models here fulfilled the goodness-of-fit test according to Hosmer and Lemeshow (*p* > 0.05). The significance level was set at 0.05 (2-sided tests). All analyses were conducted using SAS 9.4.

## Results

### Basic characteristics and correlates of stress at baseline

Having had no period of stress during the last five years was more common among 50- compared to 38-year-old women but the difference was not significant (Table 1). The mean values of sleep quality and general well-being scores were lower among women reporting higher stress levels. We applied ordinal logistic regression with stepwise variable selection to determine which baseline variables were associated with a higher level of mental stress in a mutually adjusted model. Higher age was marginally associated with less mental stress, while well-being was most strongly associated with lesser stress, OR = 0.56 (CI 0.47, 0.66) per unit of the well-being scale (Table S2). Low LTPA more than doubled the odds for more stress compared to moderate activity, while competitive sports were marginally associated with more stress.

**Table 1.** Baseline characteristics and previous and incident sick leave status by category of self-reported mental stress (*n* = 502)^a^.

		Self-reported mental stress			
	n missing	None (*n* = 129)	Sometimes (*n* = 303)	Permanent (*n* = 70)	*p*-value ^b^
Age 50 (ref = 38)	–	83 (64%)	155 (51%)	37 (53%)	0.05
BMI (kg/m^2^)	2	24.8 (4.6)	24.7 (4.7)	24.8 (4.3)	0.9
WHR^c^	3	0.81 (0.06)	0.81 (0.06)	0.82 (0.06)	0.8
University education	6	84 (66%)	205 (68%)	45 (65%)	0.9
SEI: high^d^		58 (46%)	128 (44%)	26 (39%)	
Medium		44 (35%)	125 (43%)	29 (43%)	
Low	22	23 (18%)	35 (12%)	12 (18%)	0.3
Living with a partner	4	97 (76%)	247 (82%)	51 (74%)	0.9
Number of children^e^	8	1.7 (1.1)	1.7 (1.0)	2.0 (1.2)	0.4
Current cigarette smoking	–	12 (9%)	26 (9%)	10 (14%)	0.4
Alcohol consumption (times/week)	1	2.4 (2.1)	2.1 (1.8)	2.0 (2.0)	0.4
LTPA: sedentary^f^	–	4 (3%)	21 (7%)	13 (19%)	
Moderate	–	47 (36%)	107 (35%)	27 (39%)	
Regular	–	68 (53%)	147 (49%)	23 (33%)	
Competitive sports	–	10 (8%)	28 (9%)	7 (10%)	0.004
Sleep quality^g^	5	3.9 (1.2)	3.7 (1.3)	3.0 (1.6)	0.0003
Wellbeing^g^	5	4.3 (1.0)	3.9 (1.2)	2.9 (1.2)	<0.0001
Sick leave: before baseline	–	11 (9%)	47 (16%)	19 (27%)	0.0006
after baseline	–	13 (10%)	45 (15%)	17 (24%)	0.01

^a^ mean value (SD) for continuous variables, *n* (%) for categorical variables.

^b^ Kruskal-Wallis test (continuous variables), Cochran-Armitage test of trend (binary variables), χ^2^-test (categorical variables).

^c^ Waist-to-hip ratio, a measure of fat distribution.

^d^ Socioeconomic index, based on the maximum of the woman’s and her partner’s employment, and categorised as high, medium and low.

^e^ children at home or outside home.

^f^ Leisure time physical activity, during the last year. Moderate > 4h/week.

^g^ seven categories, higher is better.

### Association between periods of mental stress at baseline and incident sick leave

Overall, 77 women experienced at least one period of sick leave before baseline (15%), and 75 women were on sick leave after baseline. Previous sick leave more than doubled the odds for incident sick leave, OR = 2.58 (1.45, 4.59), but only 21 out of 131 women on sick leave during the entire period (16%) were on sick leave both before and after baseline. Having been on sick leave before baseline (previous sick leave) was more common among women in higher stress categories. The prevalence of incident sick leave also increased across baseline stress categories, (Table 1).

Associations between mental stress at baseline and incident sick leave were estimated in three models with increasing covariate adjustment (Table 2). Model 1 shows that permanent stress during the last years tripled the risk for incident sick leave. An intermediate stress level or higher age were associated with higher odds for sick leave, but not significantly so. Previous sick leave doubled the risk for incident sick leave, but the association with permanent stress was still present (model 2). Further adjustment for variables associated with mental stress, i.e. well-being, sleep quality, and LTPA reduced the association between permanent stress and sick leave although the effect size was still large (model 3). It may be noted that the odds ratios for stress and previous sick leave hardly changed in the subset of observations with non-missing values for well-being, sleep quality, and LTPA (not shown). Further adjustment for other variables listed in Table 1 did not affect the results shown in Table 2. Using identical 1-year follow-up for all participants lowered the number of incident periods of sick leave from 75 to 60, as episodes after one year were not counted, but this did not reduce the associations described in Table 2 (not shown).

**Table 2.** Associations between stress at baseline and incident sick leave^a^.

	Model 1	Model 2	Model 3
Events / total	75 / 502	75 / 502	75 / 496
No stress (ref)	1	1	1
Some stress	1.73 (0.89, 3.37)	1.63 (0.83, 3.18)	1.49 (0.75, 2.96)
Permanent stress	3.22 (1.44, 7.21)**	2.75 (1.21, 6.26)*	2.06 (0.84, 5.02)
Age 50 (ref = 38)	1.67 (0.99, 2.80)	1.64 (0.97, 2.76)	1.59 (0.93, 2.69)
Previous sick leave	–	2.33 (1.29, 4.21)**	2.06 (1.11, 3.82)*
Wellbeing	–	–	0.96 (0.75, 1.23)
Sleep quality	–	–	0.86 (0.70, 1.05)
LTPA: low (ref)	–	–	1
Moderate	–	–	0.59 (0.25, 1.40)
Regular	–	–	0.46 (0.19, 1.10)
Competitive sports	–	–	0.42 (0.12, 1.46)
AUROC^b^	0.62	0.64	0.68

* *p* <.05, ** *p* <.01.

^a^ Odds ratio (95% CI) from logistic regression of sick leave on date of examination (effect estimate not shown), age, and mental stress (model 1), and further adjusted for previous sick leave (model 2) as well as covariates associated with mental stress (model 3).

^b^ Area under the ROC Curve, performance measurement for the classification problems at various threshold settings.

### Associations between mental stress, work-related stress and sick leave

Almost all individual items of the WSQ as well as all four domain variables were positively associated with mental stress (Table 3). Logistic regression with stepwise variable selection showed that *conflicts at work* as well as *low influence on decisions* were the strongest predictors of incident sick leave in an age-adjusted model (model 1, Table 4). These associations largely remained after further adjustment for previous sick leave and mental stress (model 2). Further adjustment for well-being, sleep quality, and LTPA, hardly changed the effect sizes for stress-related exposures but only the associations with *conflicts at work* and previous sick leave remained statistically significant (models 3). Among mean scores, *indistinct organisation and conflicts* showed the largest association with incident sick leave, OR = 2.19 (1.31, 3.67), adjusted for age and date of examination (AUROC = 0.61). Overall, regression models including specific items for work-related stress predicted incident sick-leave better than those based on mean scores (data not shown).

**Table 3.** Characteristics of work-related problems by stress level^a^.

			Self-reported mental stress			
			None (*n* = 126)	Sometimes (*n* = 298)	Permanent (*n* = 69)	
		Median	n (%)	n (%)	n (%)	p-value^b^
*Influence at work*						
s1	Lack of time to finish assignments	2	13 (10)	59 (20)	20 (29)	0.001
s2	No influence on decisions	2	39 (31)	93 (31)	28 (41)	0.3
s3	Superior’s disregard for employee’s views	2	9 (7)	40 (14)	16 (24)	0.002
s4	No control over work schedule	2	25 (20)	84 (28)	26 (38)	0.007
*q1*	*Median score*	*2*	*20 (16)*	*84 (28)*	*27 (39)*	0.0003
*Indistinct organisation and conflicts*						
s5	Increasing workload	2	27 (21)	123 (41)	44 (64)	<0.0001
s6	Unclear goals	1	31 (25)	111 (37)	34 (50)	0.0003
s7	Unclear assignments	1	13 (10)	51 (17)	19 (28)	0.002
s8	Unclear leadership	1	13 (10)	39 (13)	9 (13)	0.5
s9	Conflicts at work	1	47 (37)	153 (51)	43 (62)	0.0004
s10	Own involvement in conflicts	1	14 (11)	53 (18)	24 (35)	0.0001
s11	Superior not solving conflicts	1	25 (20)	89 (30)	25 (37)	0.008
*q2*	*Median score*	*1*	*14 (11)*	*78 (26)*	*26 (37)*	<0.0001
*Individual demands and commitment*						
s12	High demands on oneself	2	20 (16)	110 (37)	45 (65)	<0.0001
s13	Dedication to one’s work	2	12 (10)	63 (21)	30 (44)	<0.0001
s14	Work-related thoughts after work	2	12 (10)	86 (29)	29 (42)	<0.0001
s15	Difficult to set boundaries	2	35 (28)	132 (44)	42 (61)	<0.0001
s16	High responsibility	1	42 (33)	157 (53)	42 (62)	<0.0001
s17	Over-time	2	11 (9)	82 (28)	25 (36)	<0.0001
s18	Sleep problems	1	43 (34)	150 (51)	46 (67)	<0.0001
*q3*	*Median score*	*2*	*11 (9)*	*90 (30)*	*37 (53)*	<0.0001
*Interference with leisure time*						
s19	Difficult to find time for family	2	23 (18)	69 (23)	32 (46)	0.0001
s20	Difficult to find time for friends	2	28 (22)	83 (28)	36 (52)	<0.0001
s21	Difficult to find time for hobbies	2	27 (21)	91 (31)	39 (57)	<0.0001
*q4*	*Median score*	*2*	*25 (20)*	*80 (27)*	*36 (51)*	<0.0001
*other work-related variables*						
	Self-employed		5 (4)	6 (2)	3 (4)	0.9

^a^ Number (%) of participants with values larger than the median.

^b^ Cochran-Armitage test of trend.

**Table 4.** Associations between specific aspects of work-related stress and incident sick leave among women working at baseline^a^.

	Model 1	Model 2	Model 3
Events / total	75 / 492	75 / 492	75 / 490
Conflicts at work	2.31 (1.36, 3.91)**	2.05 (1.19, 3.52)**	1.98 (1.14, 3.46)*
No influence on decisions	1.77 (1.06, 2.95)*	1.71 (1.02, 2.88)*	1.71 (0.99, 2.93)
Age 50 (ref = 38)	1.52 (0.90, 2.55)	1.57 (0.92, 2.66)	1.50 (0.88, 2.57)
Previous sick leave	–	2.22 (1.21, 4.08)*	2.26 (1.18, 4.34)*
No stress (ref)	–	1	1
Some periods of stress	–	1.45 (0.73,2.87)-	1.36 (0.67, 2.72)
Permanent stress	–	2.17 (0.93, 5.06)-	1.78 (0.72, 4.40)
Wellbeing	–	–	1.02 (0.80, 1.32)
Sleep quality	–	–	0.93 (0.75, 1.14)
LTPA: low (ref)	–	–	1
Moderate	–	–	0.54 (0.22, 1.30)
Regular	–	–	0.39 (0.16, 0.95)*
Competitive sports	–	–	0.34 (0.10, 1.20)
AUROC	0.66	0.71	0.72

^a^ Odds ratio (95% CI) from logistic regression of sick leave on date of examination (effect estimate not shown), age, and specific aspects of work-related stress (model 1), and further adjusted for previous sick leave and mental stress (model 2) as well as mental stress and covariates associated with mental stress (model 3).

* *p* < 0.05, **p < 0.01.

## Discussion

### Principal findings

This study examined self-assessed mental stress as well as specific aspects of work-related stress in a population-based sample of working women aged 38 and 50 and investigated the association between these risk factors and incident sick leave of more than 14 days. Women experiencing permanent stress at baseline had a three times higher risk for incident sick leave compared to women reporting no stress. This excess risk was also observed when adjusting for previous sick leave that per se doubled the risk for incident sick leave. Specific aspects of work-related stress were also associated with incident sick leave, i.e. *conflicts at work* as well as *no influence on decisions*, and these associations were independent of previous episodes of sick leave and mental stress. The association between *conflicts at work* and sick leave was not explained by wellbeing, sleep quality and LTPA, whereas the association with *no influence on decisions* was no longer statistically significant, but the effect size prevailed. Overall, models with WSQ items predicted incident sick leave slightly better than models with general mental stress, and mental stress was not associated with sick leave in models accounting for work-related stress.

### Interpretation of results and comparison with literature

Our results show that low decision latitude (*no influence on decisions*) but not factors related to demands or workload, e.g. *individual demands on oneself* or *increasing workload,* were associated with incident sick leave. These findings are largely consistent with previous results showing that lack of control is more important for the individual workers’ well-being than high demands, as formulated using Karasek’s job demand-control model [32]. To be noted, there was no exact match between the work-related stress questions used here (WSQ) and the questions of Karasek’s model, especially regarding questions on job strain and demands. Our study also adds a new finding, namely that conflicts at work increase the risk for sick leave independent of whether or not the worker is directly involved. Gender segregation in the workplace, especially in female-dominated occupations like healthcare and education, is linked to higher sick leave rates for both women and men [16]. This may be due to the need for a positive psychosocial work climate without unresolved conflicts, which is crucial in these professions.

Previous studies have consistently supported the relationship between self-assessed mental and work-related stress and concurrent sick leave [10–12,19], which are now prospectively confirmed in the present study. In addition, we show that work-specific risk factors predict subsequent sick leave better than mental stress per se.

Previous studies have demonstrated that individuals with high demands on themselves and strong work commitment are at an increased risk of sick leave [10,32,33]. However, our study shows that these individual factors appear to be less influential in predicting sick leave compared to workplace characteristics, i.e. low decision latitude and conflicts at work.

### Implications for research and practice

The primary findings from this study underscore a robust association between baseline self-reported mental and work-related stress, with future sick leave in gainfully employed middle-aged women. Conducted as a population study rather than involving patients already on sick leave, this research provides insights for employers, occupational health care and primary care on how to address mental and work-related stress in women before it escalates to sick leave.

Both women and men seek primary health care for various physical and mental symptoms, and often in an early stage, long before a sick leave is relevant [34,35]. These symptoms of ill health can be related to stress in general or work-related. Neither the patient nor the GP might be aware of the fact that the symptoms of ill health are related to factors at work [11, 36,37]. Our study reveals specific work-related problems such as conflicts at work and low decision influence as the strongest predictors of incident sick leave. In the Swedish context, the employer is responsible for the overall working environment but also for, if necessary, improving the working situation for the individual employee [38]. However, not only the fact that people go to primary care for their various symptoms, primary health care in Sweden also has a preventive mission through legislation [39]. This calls for the development of interventions in primary health care that early identify patients with work-related stress and offer preventive measures.

It is important to consider the interaction between the individual and the work environment, which means interventions that involve collaboration both internally between various professions and externally with other stakeholders. This underscores the importance of addressing workplace conflicts and enhancing decision influence in mitigating sick leave due to stress-related issues, where perceived mental stress is a risk factor for future sick leave. It is essential to acknowledge the multifaceted nature of stress and well-being. Potential interventions may encompass psychosocial support mechanisms but also workplace collaborations between primary health care and the social insurance agency on the one hand and employers and occupational health care on the other hand [40] to mitigate stress and improve overall well-being and risk for sick leave. Further research could use focus groups to understand women’s perceptions of stress better. It is also important to complement the current findings from middle-aged women with conclusions from other demographic groups (e.g. younger women and middle-aged men) to understand potential gender and age differences in the perception of stress and its impact on sick leave.

In our study, engaging in low levels of leisure-time physical activity more than doubled the likelihood of experiencing higher self-reported stress levels compared to those who engage in moderate or regular physical activity. Research indicates that individuals who do not participate in enough physical activity during their leisure time are more prone to experiencing higher stress levels at work [41]. It is crucial to raise awareness about the link between low levels of leisure-time physical activity and increased self-reported stress. This association might be due to stress interfering with health-promoting behaviours or an unhealthy lifestyle leading to a greater perception of stress. It is likely that both factors are involved.

Our findings also support the idea that leisure-time physical activity has a protective effect against sick leave. However, while some employers encourage their employees to be physically active, it is important to remember that this cannot substitute for the ongoing assessment of the work environment and addressing its issues.

### Strengths and limitations

The study’s strengths included the population-based random sample with a high participation rate and the use of validated questionnaires for stress exposures, well-being and sleep quality. The study followed the participants over time, allowing for the assessment of changes and outcomes in sick leave before and after the baseline examination. Another strength was the objective measurement of sick leave by registry data (MiDAS). One limitation of the study was the sample size. A larger sample size might have enabled a more comprehensive analysis of specific work-related factors and potential confounders. Another limitation is the lack of information from the hospital registry that would have allowed a more specific outcome definition by excluding sick leave episodes due to non-work-related causes. The generalisability of the findings to other countries might be limited as entitlement to sick leave varies depending on the country and employer policies [42,43].

## Conclusion

Work-related stress more than doubled the risk for incident sick leave independent of mental stress and previous periods of sick leave. People primarily seek primary care for their early stress-related symptoms. Therefore, it is reasonable to develop interventions to prevent long-term ill health and sick leave. Further, it is important to develop a basis for collaborations with relevant stakeholders.

## Data Availability

The data sets used and analysed during the current study are available from the corresponding author upon reasonable request.
